# Supplementary material for: Methylome evolution suggests lineage-dependent selection in the gastric pathogen Helicobacter pylori
Source: Commun Biol. 2023 Aug 12;6:839. doi: 10.1038/s42003-023-05218-x (PMC10423294; doi:10.1038/s42003-023-05218-x)
Supplement: Supplementary file 2 — Supplementary Information [file 42003_2023_5218_MOESM2_ESM.pdf]

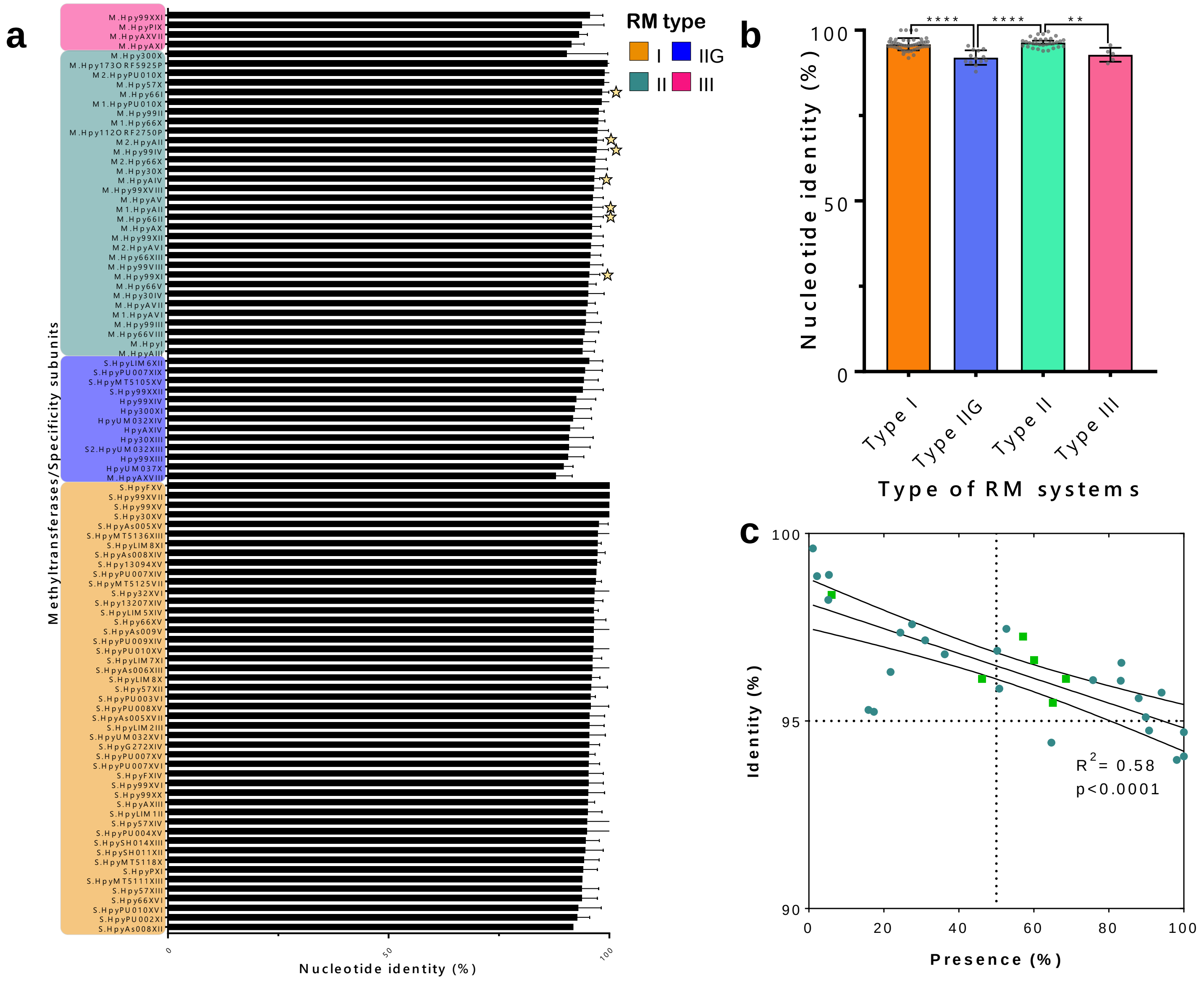

Supplementary Figure 1 – Sequence conservation of methyltransferases in *H. pylori*. **a**. Nucleotide diversity of 96 methyltransferases and specificity subunits. Type II methyltransferases flanked by direct repeats are indicated by a star. Error bars represent 95% confidence intervals. **b**. Average identity in each type (Dunn's test two-sided **** p < 0.0001, ** p < 0.01). Error bars represent 95% confidence intervals. **c**. Scatter plot of the frequency and nucleotide identity of type II methyltransferases. Methyltransferases flanked by direct repeats are indicated by a square symbol. The correlation coefficient obtained by linear regression is indicated.

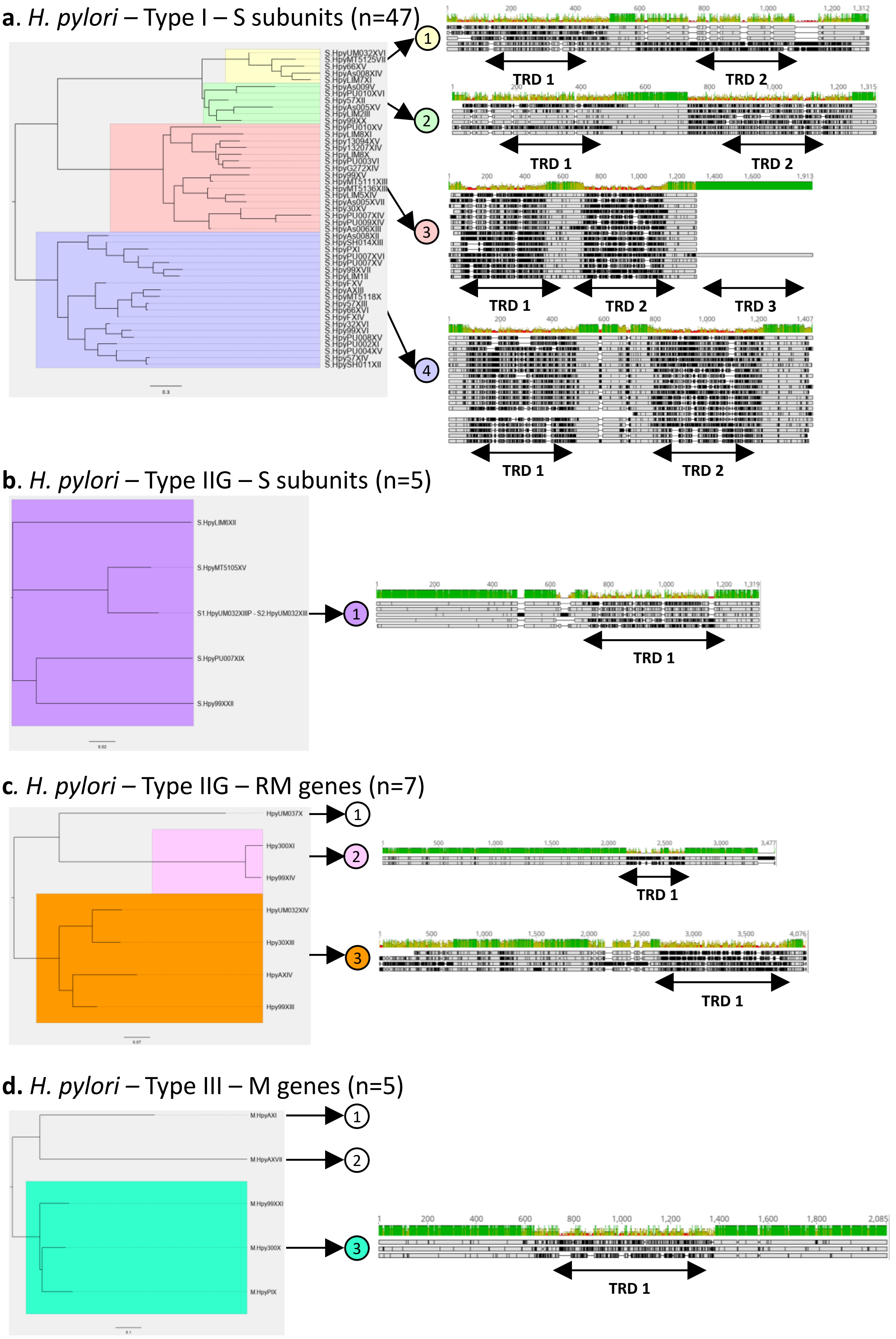

Supplementary Figure 2 – Phylogeny of type I, IIG, and III RM systems in *H. pylori*. Clusters of methyltransferases determined by phylogeny and confirmed by inspection of multiple alignments are indicated by colour boxes. For each cluster, a visual representation of the multiple alignment is provided (black lines indicate positions which are different between strains). An identity diagram is located on top of each alignment and shows conserved (in green) and highly variable (in red) positions. On the bottom of the alignment, the position of target-recognition domains is indicated by horizontal arrows. The diversity of target-recognition domains within a conserved methyltransferase backbone can be appreciated by remarking the conserved positions of the backbone interspersed by clusters of variable positions from the domains. **a**. Phylogeny of S-subunits from type I RM systems. **b**. Phylogeny of S subunits from type IIG RM systems. **c**. Phylogeny of RM genes from type IIG RM systems. **d**. Phylogeny of methyltransferases from type III RM systems.

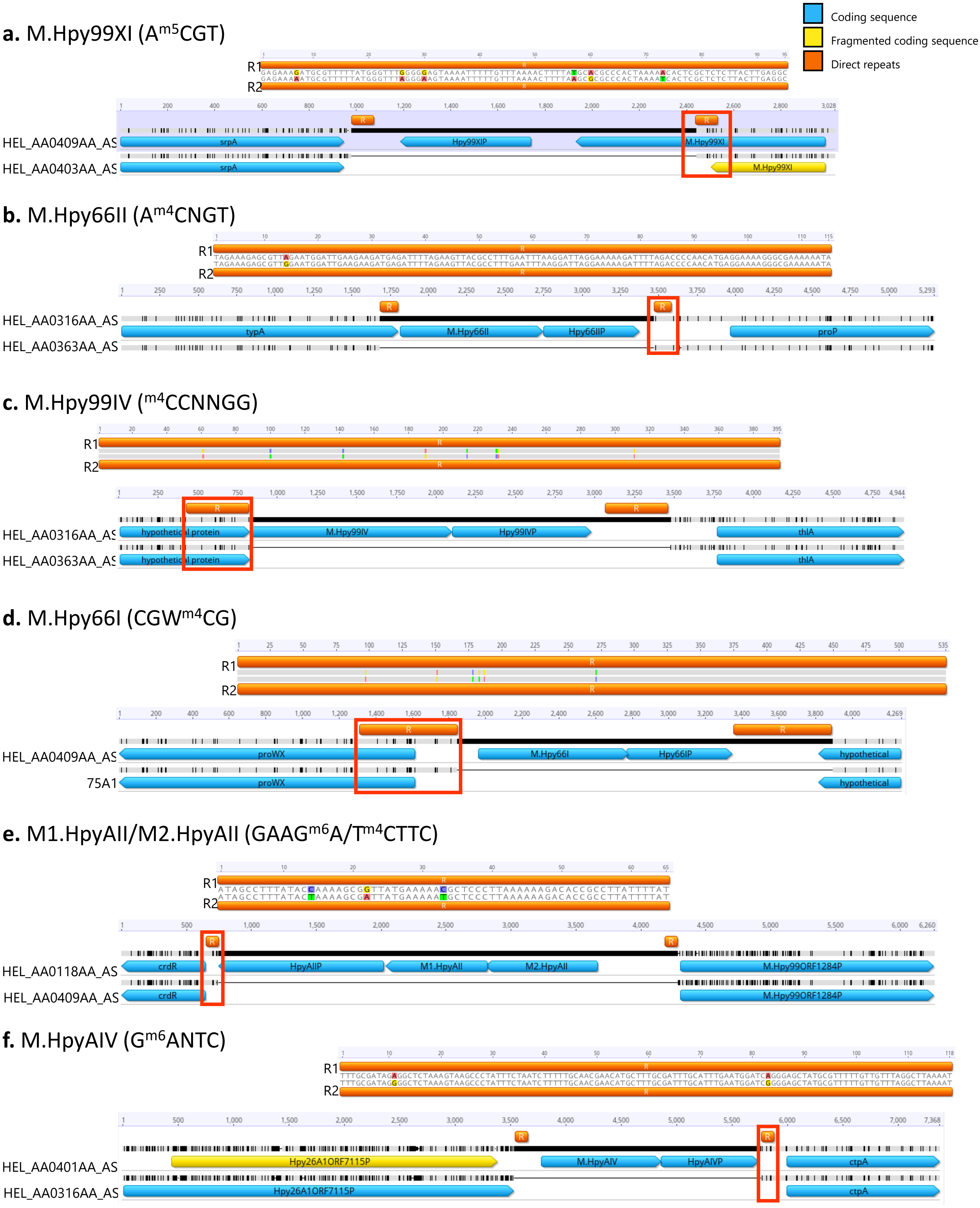

Supplementary Figure 3 – Direct repeats flanking six different type II RM systems. The genetic context in strains in which the given type II RM system is either present (top) or absent (bottom) is shown as a pairwise alignment. Complete and fragment coding sequences are coloured in blue and yellow, respectively. The direct flanking repeats are indicated by orange boxes and the repeat remaining in both strains is highlighted by a red box. An alignment of the left and right flanking repeats is also provided to visualized the similarity between them. A. M.Hpy99XI (Am^5CGT). B. M.Hpy66II (Am^4CNGT). C. M.Hpy99IV ($^{m4}CCNNGG$). D. M.Hpy66I ($CGWm^4CG$). E. M1.HpyAII/M2.HpyAII ($GAAG^{m6}A/T^{m4}CTTC$). F. M.HpyAIV ($G^{m6}ANTC$).

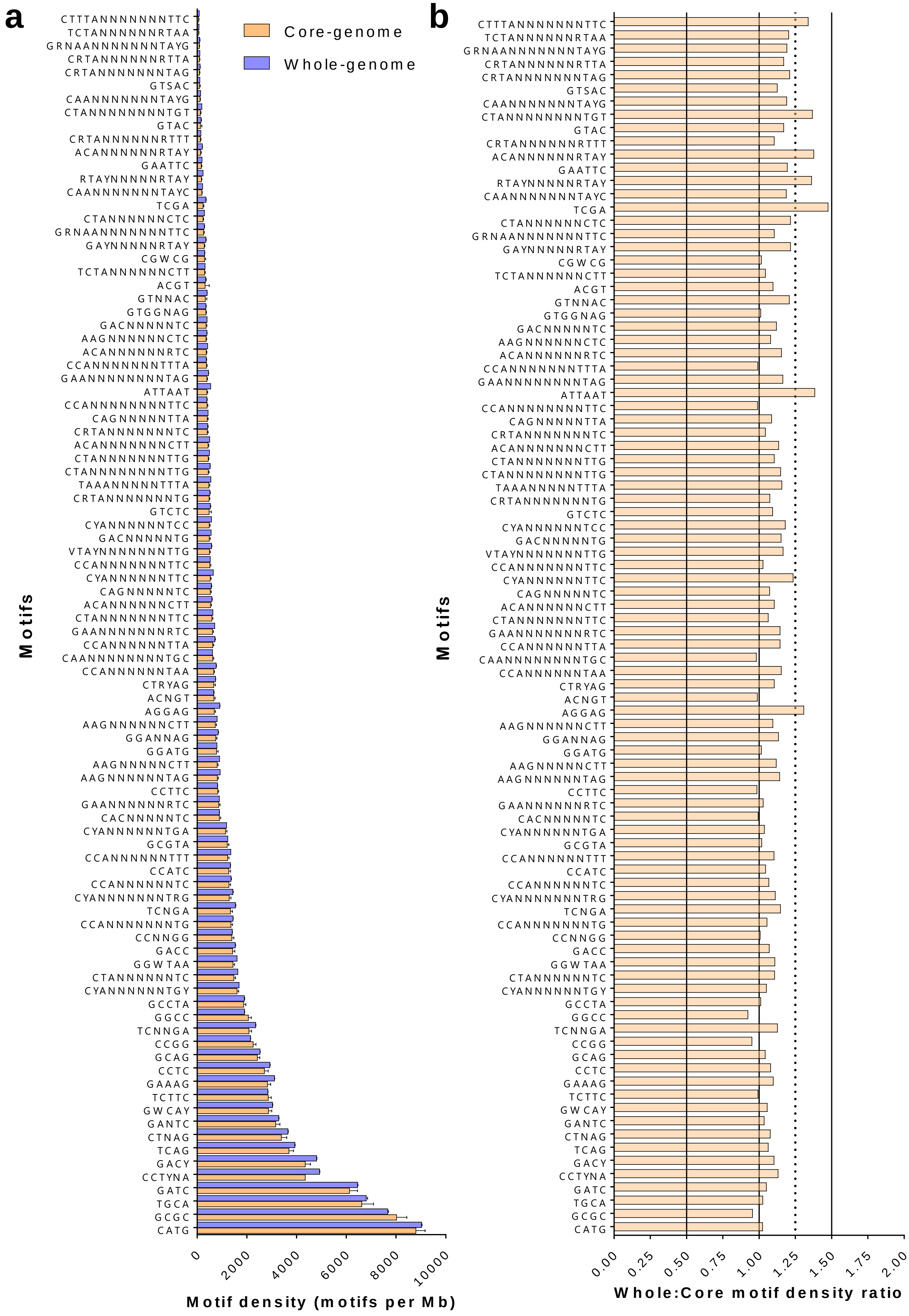

Supplementary Figure 4 – Comparison of target motif density in whole genome and core genes. **a**. Motif density (motifs/Mb) of 92 target sequences calculated in a core gene alignment. Error bars represent 95% confidence intervals. **b**. Ratio of motif density calculated on whole genomes versus core genes.

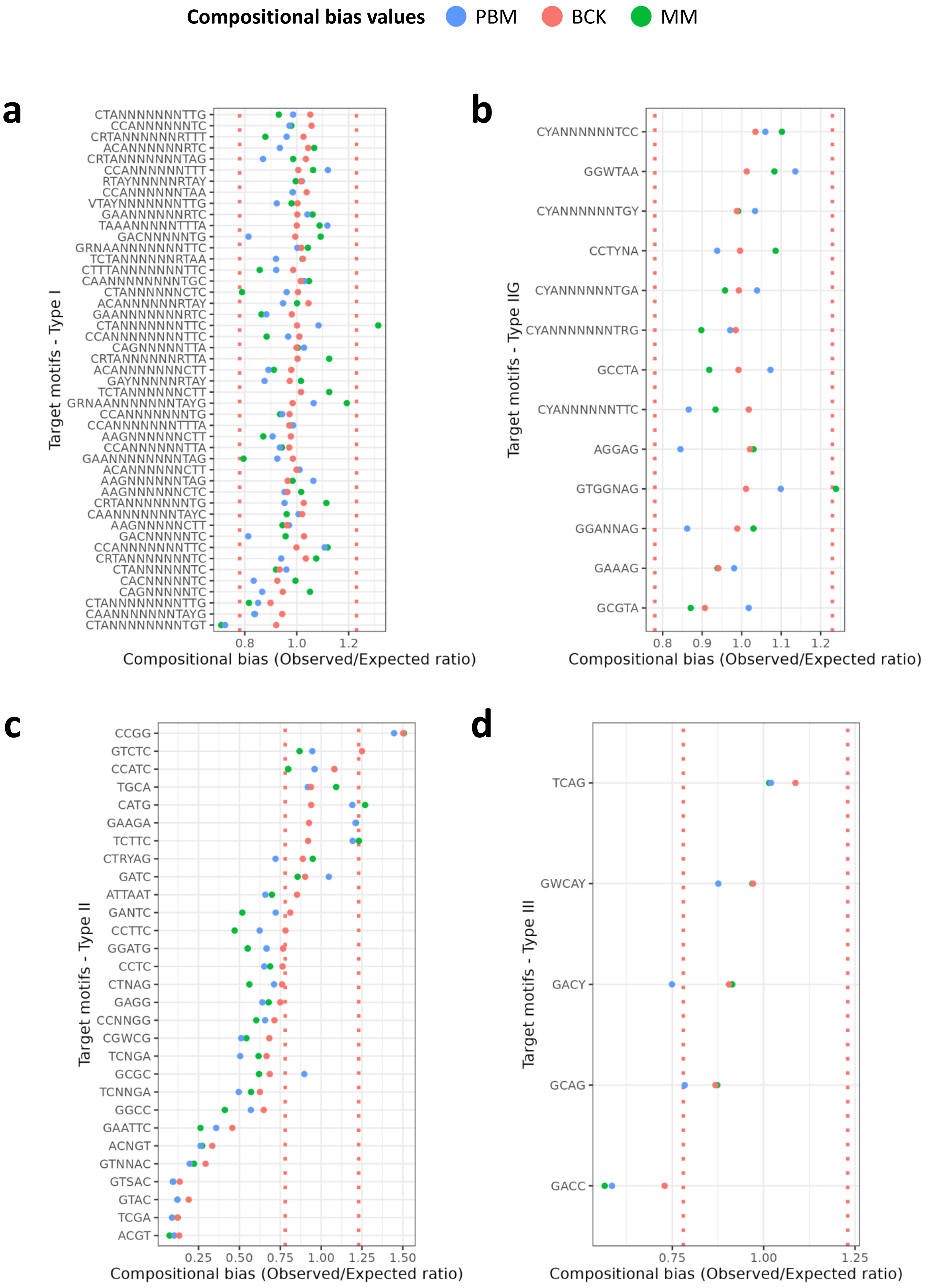

Supplementary Figure 5 – Compositional bias calculation for target motifs in *H. pylori* using three different methods. **a**. Type I motifs. **b**. Type IIG motifs. **c**. Type II motifs. **d**. Type III motifs. MM: method based on maximum order Markov chain. PBM: method based on Pevzner and co-authors. BCK: method based on Burge and co-authors. Under- and over- represented cutoffs are represented by vertical lines.

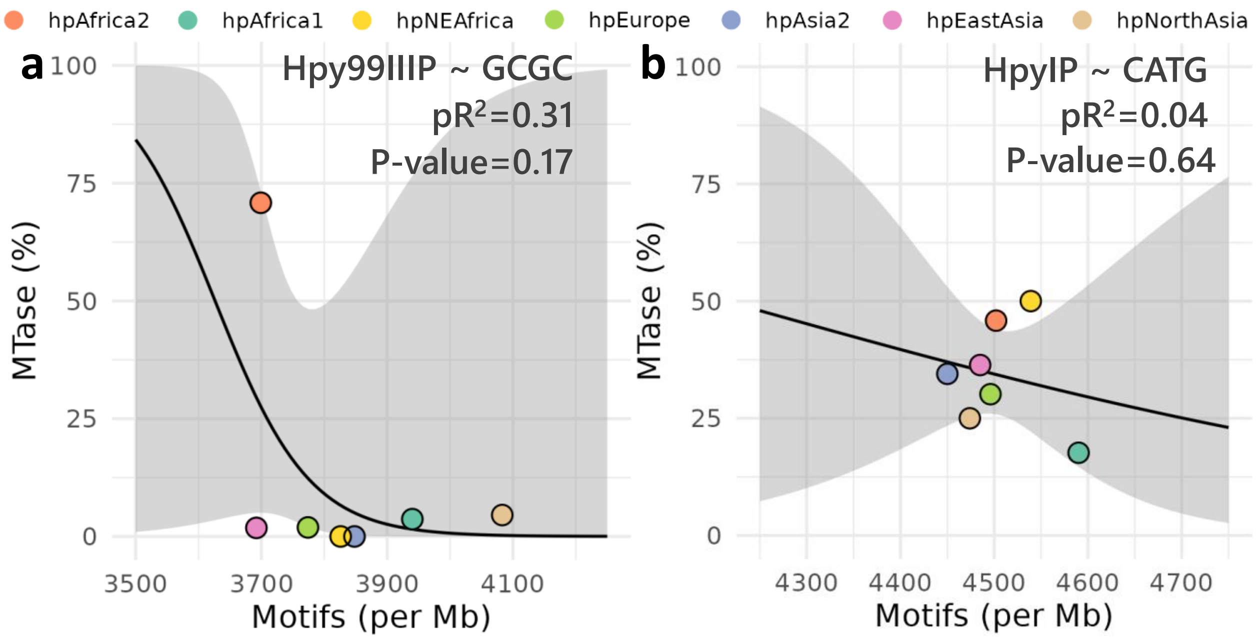

Supplementary Figure 6 – Interaction between endonuclease frequency and motif density in two type II RM systems. **a**. Hpy99IIIP **b**. HpyIP. Pseudo R^2 (calculated with the Mc Fadden method) and p-values are indicated. 95% confidence interval is indicated by a grey ribbon.

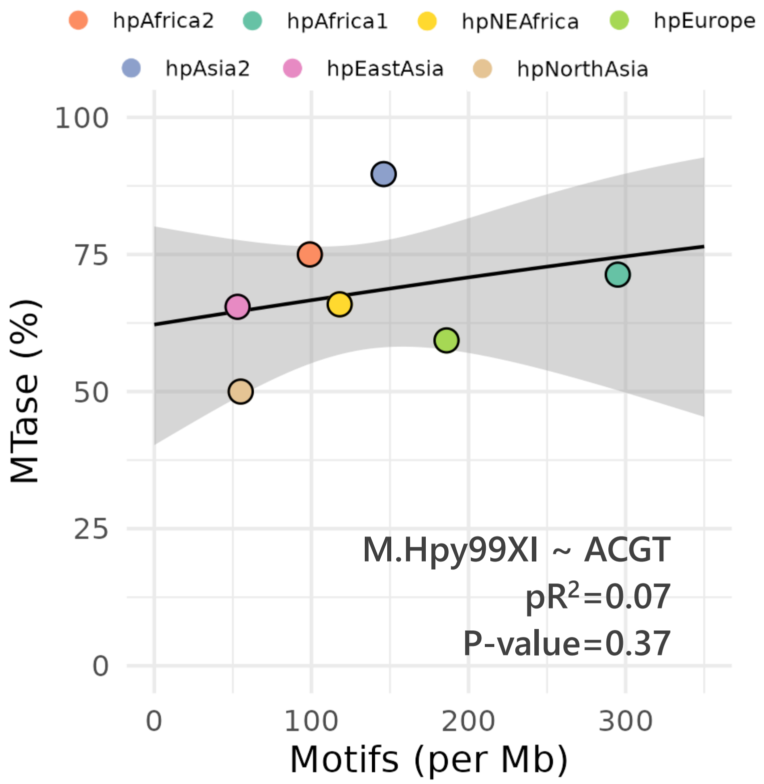

Supplementary Figure 7 – Interaction between methyltransferase frequency and motif density for the Hpy99XI RM system. Pseudo R^2 (calculated with the Mc Fadden method) and p-values are indicated. 95% confidence interval is indicated by a grey ribbon.
