# Supplementary material for: Methylome evolution suggests lineage-dependent selection in the gastric pathogen Helicobacter pylori
Source: Commun Biol. 2023 Aug 12;6:839. doi: 10.1038/s42003-023-05218-x (PMC10423294; doi:10.1038/s42003-023-05218-x)
Supplement: Supplementary file 3 — Description of Additional Supplementary Files [file 42003_2023_5218_MOESM3_ESM.pdf]

Description of Additional Supplementary Files

File Name: Supplementary Data 1
Description: Restriction-Modification systems of H. pylori. Type of RM systems, names and associated target-motifs are indicated. Average methylation rates based on PacBio data determined in *H. pylori* strains (designated by their REbase strain number) are provided.

File Name: Supplementary Data 2
Description: Genome collection of *H. pylori*. Geographic population and sub-population are indicated. Accession numbers from either Enterobase or NCBI Bioproject are provided.

File Name: Supplementary Data 3
Description: Presence/absence of 96 methyltransferases in 541 genomes of *H. pylori*. Presence and absence of the methyltransferase are indicated by 1/green and 0/red, respectively.

File Name: Supplementary Data 4
Description: Sequence of direct repeats flanking six different type II RM systems.

File Name: Supplementary Data 5
Description: Results of the generalized linear model analysis for association between motif density and RM system frequency in *H. pylori* geographical populations. Pseudo R2 (calculated with the Mc Fadden method), p-values, FDR-adjusted p-values and direction of the relationship (positive or negative) are indicated.

File Name: Supplementary Data 6
Description: Comparisons of methyltransferase frequencies based on blastn versus blastp alignments.

File Name: Supplementary Data 7
Description: Quality control for the whole-genome consensus alignment. Pairwise identity and mean coverage compared to the reference sequence BCM-300 are indicated.

File Name: Supplementary Data 8
Description: Source data for main figures.
